# Development of self-image and its components during a one-year follow-up in non-referred adolescents with excess and normal weight

**DOI:** 10.1186/s13034-015-0038-7

**Published:** 2015-02-26

**Authors:** Mauno Mäkinen, Mauri Marttunen, Erkki Komulainen, Viacheslav Terevnikov, Leena-Riitta Puukko-Viertomies, Veikko Aalberg, Nina Lindberg

**Affiliations:** Adolescent Psychiatry, University of Helsinki and Helsinki University Hospital, Helsinki, Finland; National Institute for Health and Welfare, Helsinki, Finland; Behavioural Sciences, University of Helsinki, Helsinki, Finland; Forensic Psychiatry, University of Helsinki and Helsinki University Hospital, Helsinki, Finland; Children’s Hospital, University of Helsinki and Helsinki University Hospital, Helsinki, Finland

**Keywords:** Self-image, Adolescence, Excess weight, Normal weight

## Abstract

**Background:**

The proportion of overweight and obese youths is high. The present study aimed to investigate the development of self-image and its components during a one-year follow-up among non-referred adolescents with excess and normal weight. Furthermore, we separately analyzed the data for girls and boys.

**Methods:**

Altogether 86 8^th^ grades (41 girls and 45 boys) with a relative weight of 26% or more above the median and 91 controls (43 girls and 48 boys) with normal weight participated the follow-up. The Offer Self-Image Questionnaire, Revised (OSIQ-R) was used to assess self-image at baseline and on follow-up. In the OSIQ-R, a low total raw score implies positive adjustment, while a high raw score implies poor adjustment and a negative self-image. The study design was doubly correlated (pairs and time), and a linear mixed model was used in the statistical analysis.

**Results:**

In OSIQ-R total scores, a comparative improvement was observed in girls with normal weight. Among these girls, significant change scores compared to zero were seen in impulse control, social functioning, vocational attitudes, self-confidence, self-reliance, body image, sexuality, and ethical values. In girls with excess weight, none of the change scores compared to zero were statistically significant. When the girls with normal and excess weight were compared, the difference in change scores was largest in sexuality and vocational attitudes. Change scores compared to zero were significant in sexuality and idealism for boys with excess weight, and in impulse control, mental health, self-reliance, and sexuality for normal weight boys. When the boys with excess and normal weight were compared, no statistically significant differences emerged in change scores.

**Conclusion:**

In mid-adolescent girls, the influence of overweight and obesity on the development of self-image is substantial. Weight management programs directed at overweight adolescent girls should include psychological interventions aiming to diminish self-image distress, especially that associated with feelings, attitudes, and behavior towards the opposite sex, as well as future career plans.

## Background

Adolescence is a period of life when individuals transfer from childhood and their biological, cognitive, psychological, and social characteristics become more adult-like. The key developmental tasks of adolescence are the achievement of biological and sexual maturity, the development of personal identity, the development of intimate sexual relationships, and finally, the establishment of independence and autonomy [1]. Adolescence is an intensive period in which the salience of body shape is considerable. New roles are negotiated in areas that are tied to physical appearance [2,3].

Overweight and obesity have become a global epidemic among adolescents of all ethnic and socioeconomic backgrounds. Excess weight is linked to diminished subjective well-being, including a poor quality of life [4], body dissatisfaction [5], low self-esteem [6], poor academic performance [7], depression [8], high levels of sadness and anxiety [9], and eating disorder pathology [10]. Furthermore, overweight adolescents are described as socially marginalized; they are at greater risk of mistreatment by peers and have fewer opportunities to develop intimate romantic relationships [11]. Research on adolescent community samples, however, has suggested that despite moderate levels of body dissatisfaction, relatively few adolescents with excess weight show low self-esteem or psychiatric comorbidity, or report poor emotional or social functioning [12]. Furthermore, findings from a large community survey demonstrated no association of body mass index (BMI) with eating disorders [13]. According to a follow-up community study by Roberts and Hao [14], obesity has limited effects on the future psychosocial functioning of adolescents. The authors, for example, found no evidence that academic performance is negatively impacted by obesity.

Self-image, according to Offer et al. [15], can be regarded as the organization of an individual’s perception of functioning and adjustment in different areas of his or her life. It is a multidimensional construct with different aspects described as the psychological, social, sexual, familial, and coping self. This reflects the necessity to evaluate adolescent functioning in multiple areas, because it is possible to master certain areas while having difficulties in others. In healthy youngsters, the change in self-image from early to mid-adolescence is generally positive [16]. A negative self-image has been associated with many psychological problems such as low self-esteem [17], problems at school [18], depression [19], and eating disorders [20-23], as well as with overweight and obesity [24]. Concerning the components of self-image, overweight girls have been reported to be less adjusted with respect to their sexual attitudes and to present more psychopathology than their peers with average weight [25]. Moreover, distortion of the body image has been linked to overweight and obesity [26,27]. According to a recent study by Farhat et al. [28], body image mediated the relationship of obesity with infrequent breakfast consumption in both genders, but among girls also with smoking and a lack of physical activity. Furthermore, body image had a stronger association with victimization and bullying than objective BMI-derived weight classification [29]. According to Roberts and Duong [30], perceived weight rather than obesity increases the risk of major depression among adolescents.

Among adults, there is some evidence that subjective well-being variables influence success in weight loss [31], and a greater focus on these variables both in obesity prevention and weight management programs has been demanded [32]. Furthermore, among adolescents, emotional correlates of excess weight are important to assess in order to target individually appropriate interventions that could enhance well-being [33]. One way to obtain more information on adjustment problems associated with overweight and obesity in adolescence is to study self-image and its development. The results might shed light on the important question of what types of psychosocial interventions should be included in weight management programs directed at adolescents. Previous self-image studies have hinted that problems might exist in the areas of psychological, sexual, and coping self among youngsters with excess weight, especially girls. However, as earlier research focusing on the psychosocial well-being of overweight and obese adolescents has been characterized by highly contradictory results, more research is needed, especially in the form of follow-up studies.

The aim of the present study was to investigate the development of self-image and its components during a one-year follow-up period among non-referred adolescents with excess and normal weight. Furthermore, as gender-specific differences exist in the self-image and its components [34,35], we separately analyzed the data for girls and boys.

## Materials and methods

### Participants

The study subjects were 2499 adolescent girls and boys attending the 8th grade at 24 out of 70 (34%) secondary schools in the city of Helsinki who agreed to participate in the study project in 2003 and 2004 [36]. Although the general population in Helsinki is relatively homogeneous, the schools covered all the representative socio-economic groups across the city districts, including state and municipal, as well as private schools. From the 2499 students, those attending ordinary education programs and speaking Finnish as their mother tongue were selected (n = 2286, 91.5%). For 916 (40.1%) students, either the student him/herself or his/her guardians did not provide consent and they were omitted from the sample. Thus, 1370 students (659 girls and 711 boys) with a mean age of 14.5 (SD 0.3) participated in the study.

### Procedures

The adolescents completed self-assessment during their ordinary school lessons. Teachers, familiarized with the study protocol by the researchers, explained the study procedure to their students as well as delivering and collecting the self-assessment and consent forms. School nurses measured the students’ body weights and heights. Of those, who were obese or overweight (the excess-weight group), those, who agreed to attend the one-year follow-up study were selected. They as well as their control subjects completed the self-assessment and the school nurses measured their weights and heights in both the initial phase and at the one-year follow-up.

### Measures

#### Self-image

The Offer Self-Image Questionnaire, Revised (OSIQ-R) [37] was used to assess self-image at baseline and on one-year follow-up. The OSIQ-R is a 129-item objective personality test for 13- to 18-year-old adolescents measuring the feelings of teenagers about their own psychological world. Self-image is conceptualized as a multidimensional construct in the OSIQ-R. Therefore, the OSIQ-R is designed to encompass 12 dimensions, referred to as component scales. Each component scale corresponds to an aspect of functioning that is thought to be important to adolescents: emotional tone, impulse control, mental health, social functioning, family functioning, vocational attitudes, self-confidence, self-reliance, body image, sexuality, ethical values, and idealism. In addition to these 12 component scales, the overall self-image is measured by the total self-image scale, which combines scores across 10 of the component scales. The total self-image scale does not include the sexuality and idealism scales, because their correlation with the other scales is low. Ratings are evaluated using a six-point Likert scale: describes me very well (1) – does not describe me at all (6). A low total raw score implies positive adjustment, while a high raw score implies poor adjustment and a negative self-image. The OSIQ has been used and validated among Finnish adolescents [38-41]. In the present study, the adolescents completed the OSIQ-R during their regular school hours. Cronbach’s alpha was used as the reliability measure both at baseline and on follow-up (Table 1).

**Table 1:** **The Offer Self-Image Questionnaire, Revised (OSIQ-R)**

	**Girls/BL CA**		**Girls/FU CA**		**Boys/BL CA**		**Boys/FU CA**	
	**EW**	**NW**	**EW**	**NW**	**EW**	**NW**	**EW**	**NW**
	**Mean (SD)**	**Mean (SD)**	**Mean (SD)**	**Mean (SD)**	**Mean (SD)**	**Mean (SD)**	**Mean (SD)**	**Mean (SD)**
Total Self-Image Scale (113 items)	0.93		0.91		0.92		0.93	
Overall self-image	2.60 (0.56)	2.70 (0.56)	2.52 (0.51)	2.53 (0.52)	2.40 (0.48)	2.25 (0.46)	2.35 (0.50)	2.17 (0.50)
*OSIQ*-*R Component Scales*								
Emotional Tone (10 items)	0.87		0.89		0.85		0.90	
The degree of affective harmony within the structure	2.59 (0.77)	2.77 (0.82)	2.56 (0.85)	2.60 (0.83)	2.16 (0.70)	2.04 (0.61)	2.15 (0.66)	1.95 (0.61)
Impulse Control (9 items)	0.75		0.72		0.71		0.79	
The extent to which an adolescent’s egois strong enough to handle various pressures without resorting to unacceptable tension-discharging actions	2.77 (0.67)	2.89 (0.62)	2.63 (0.61)	2.68 (0.59)	2.33 (0.58)	2.22 (0.55)	2.32 (0.59)	2.08 (0.63)
Mental Health (13 items)	0.84		0.77		0.74		0.77	
Emotional health in terms of the relative absence of psychopathological thought processes	2.49 (0.71)	2.55 (0.72)	2.43 (0.69)	2.38 (0.58)	2.27 (0.56)	2.15 (0.60)	2.16 (0.56)	2.00 (0.57)
Social Functioning (9 items)	0.83		0.82		0.76		0.77	
Patterns of interpersonal relationships and friendships	2.52 (0.85)	2.51 (0.79)	2.47 (0.89)	2.33 (0.62)	2.30 (0.62)	2.16 (0.60)	2.24 (0.72)	2.10 (0.53)
Family Functioning (19 items)	0.92		0.90		0.88		0.88	
An adolescent’s feelings about, and relationships with, his or her parents	2.35 (0.77)	2.59 (0.82)	2.26 (0.70)	2.51 (0.77)	2.28 (0.63)	2.11 (0.65)	2.26 (0.62)	2.10 (0.63)
Vocational Attitudes (10 items)	0.67		0.69		0.69		0.76	
The degree of confidentiality an adolescent feels in learning about and planning for a vocation	2.36 (0.56)	2.49 (0.52)	2.39 (0.54)	2.32 (0.55)	2.24 (0.55)	2.12 (0.47)	2.22 (0.62)	2.11 (0.53)
Self-Confidence (10 items)	0.77		0.77		0.71		0.83	
An adolescent’s capacity to adapt to his/her immediate environment	2.66 (0.64)	2.67 (0.64)	2.57 (0.62)	2.52 (0.68)	2.43 (0.63)	2.27(0.53)	2.36 (0.64)	2.15 (0.64)
Self-Reliance (14 items)	0.59		0.61		0.55		0.73	
An adolescent’s ability to cope with himself or herself, other people, and his or her own world	2.97 (0.51)	3.00 (0.45)	2.92 (0.51)	2.86 (0.46)	2.77 (0.50)	2.72 (0.44)	2.75 (0.55)	2.56 (0.60)
Body image (9 items)	0.81		0.81		0.83		0.86	
The extent to which an adolescent has adjusted to his or her body	3.08 (0.79)	2.95 (0.76)	2.88 (0.75)	2.64 (0.75)	2.64 (0.76)	2.08 (0.62)	2.56 (0.80)	2.05 (0.66)
Sexuality (10 items)	0.79		0.74		0.73		0.67	
An adolescent’s feelings, attitudes, and behavior towards to opposite sex	2.78 (0.74)	2.84 (0.54)	2.66 (0.73)	2.50 (0.47)	2.47 (0.63)	2.22 (0.56)	2.31 (0.56)	2.07 (0.45)
Ethical values (10 items)	0.52		0.54		0.65		0.73	
The extent to which the conscience has developed	2.51 (0.60)	2.62 (0.53)	2.44 (0.63)	2.44 (0.46)	2.59 (0.60)	2.50 (0.62)	2.49 (0.66)	2.46 (0.67)
Idealism (6 items)	0.54		0.62		0.58		0.62	
An adolescent’s ideals and his or her willingness to help others	3.06 (0.62)	3.25 (0.61)	3.13 (0.59)	3.20 (0.72)	3.27 (0.69)	(0.71)	3.50 (0.73)	3.47 (0.71)

A short description of the scales. Cronbach’s alpha (CA) was used as the reliability measure at baseline (BL) and on follow-up (FU) among girls (n = 78) and boys (n = 88). Means and standard deviations (SD) of the OSIQ-R scores are shown among girls with excess (n = 38) and normal weight (n = 40) and among boys with excess (n = 42) and normal weight (n = 46).

#### Weight

The BMI and relative weight were calculated, and the results were used to reflect the degree of excess body weight. Previous research has indicated that the reference values increase with age, and BMI may be a valid measure of adiposity among adolescents [42]. Consequently, the respective cut-off points of 25 and 30 kg/m^2^ for overweight and obesity commonly used for adults were substituted with the international lower cut-off points of BMI percentiles for adolescents [43]. In addition, the < 5th percentile of the reference curves for Finnish children was used as a cut-off point for being underweight [44]. According to the Finnish Current Care Guidelines for obesity in children [45], the relative weight or weight-for-height represents the percentage deviation of the weight from the median value for any given height according to gender. A relative weight 15.0% or more under the median weight was considered as underweight, a relative weight 20-40% higher than the median as overweight, and a relative weight over 40% higher than the median weight as obesity. Accordingly, of the 1370 students, 97 (7.1%; 49 girls and 48 boys) were underweight, 1027 (75.0%; 498 girls and 529 boys) of normal weight, 141 (10.3%; 68 girls and 73 boys) overweight, and 43 (3.1%; 16 girls and 27 boys) obese. Weight and/or height data were missing for 62 adolescents (4.5%; 28 girls and 34 boys). According to BMI, the respective values were, 55 (4.0%; 28 girls and 27 boys) underweight, 1076 (78.5%; 529 girls and 547 boys) of normal weight, 144 (10.5%; 65 girls and 79 boys) overweight, and 33 (2.4%; 9 girls and 24 boys) obese.

In the present study, all adolescents with a relative weight of 26% or more above the median comprised the group of adolescents with excess weight (n = 114, 8.3%; 53 girls and 61 boys). Altogether 92 adolescents with excess weight agreed to participate the follow-up study and the controls matched by sex-, age (+/− 3 months), school and weight (relative weight of +/− 5% within the median weight for the respective gender cohort) were selected for them. The weights and heights were re-measured (the baseline of the follow-up) by the school nurses, which revealed that five of the adolescents with excess weight (2 girls and 3 boys) had moved to normal weight according to their relative weight or BMI. They were excluded from the follow-up. Further, one boy was excluded because of lacking a valid case–control subject. So, altogether 86 adolescents with excess weight and 91 controls with normal weight started the follow-up period. During the follow-up, five adolescents with excess weight (2 girls and 3 boys) and five controls (3 girls and 2 boys) dropped out. One girl with excess weight was excluded because of 15 missing values in her self-assessment at the follow-up phase. Finally, 80 adolescents with excess weight (38 girls and 42 boys) and 86 with normal weight (40 girls and 46 boys) attended the study in both the initial phase and at the one-year follow-up. In initial phase of the study, the lowest relative weight was 25% for girls and 23% for boys. The respective values for BMI were 24.29 kg/m^2^ and 23.74 kg/m^2^. Among the girls with excess weight, the mean relative weight in the initial phase was +39.13% (SD 11.75) and the mean BMI value 27.12 kg/m^2^ (SD 2.24). Among the boys with excess weight, the respective mean values were +45.17% (SD 17.78) and 28.48 kg/m^2^ (SD 3.37). At the one-year follow-up, the mean relative weight of the girls with excess weight was +40.76 (SD 16.79) and the mean BMI value 27.47 kg/m^2^ (SD 3.29). Among the boys with excess weight, the respective mean values were +44.74 (SD 19.91) and 29.08 kg/m^2^ (SD 4.13). Among the female controls, the mean relative weight in the initial phase was +2.60 (SD 5.34) and the mean BMI value 20.02 kg/m^2^ (SD 1.06), while among the boys, the respective mean values were −0.15 (SD 4.65) and 19.80 kg/m^2^ (SD 1.16). At the one-year follow-up, the mean relative weight of the girls with normal weight was +4.13 (SD 7.06) and the mean BMI value 20.47 kg/m^2^ (SD 1.50), while among the boys with normal weight, the respective mean values were +2.13 (SD 6.08) and 20.63 kg/m^2^ (SD 1.49).

### Statistical analyses

Single missing values in the OSIQ-R were imputed using the expectation maximization method. The proportion of imputed values was 3.3‰ in the initial phase and 2.4‰ at the one-year follow-up. The independent samples *t*-test and Little’s test were used for the dropout analysis. To increase the comparability between scales, the scores were calculated by dividing the sum by the number of items in the scale. The statistical method for this type of design needs to master the dependencies that arise from matching the pairs and repeating the measures. One such technique is linear mixed model analysis (LMM) [46]. The LMM enabled us to keep the controls of those 5 adolescents who reduced their weight to normal during the screening and the second measurement (baseline), and, because of this, were excluded, in the study population. Various *post hoc* tests were run using capabilities in the LMM (the TEST procedure). All the *post hoc* tests were run using no correction, i.e. with LSD as the default. The findings were considered significant when p < 0.05. No correction, such as Bonferroni, was applied to control for type I errors due to the multiple comparisons, as it has been criticized for dramatically increasing the risk of type II errors [47-49]. Change comparisons between subgroups in component scales lacked statistical power. The statistical sensitivity also varied between the comparisons, depending upon the correlation between baseline and follow-up measures. Consequently, graphical presentations based on effect sizes are reported, as this improves the comparability of the results. Cohen’s d (d) indices were calculated by dividing the observed difference by the pooled standard deviation. For Cohen’s d, an effect size of 0.2 to 0.3 can be interpreted as a “small” effect, around 0.5 as a “medium” effect and 0.8 or above, as a “large” effect [50]. Thus, results greater than the absolute value d = 0.2 are reported. The data were analyzed using SPSS for Windows, version 22.0 [51].

### Ethics

The Ethics Committee of the Hospital for Children and Adolescents at Helsinki University Central Hospital approved the study. Letters outlining the nature of the study were sent to the parents or guardians of the under-aged participants. Either active or passive consent of parents or guardians was obtained. The participants were also requested to provide their own written permission when completing the questionnaire in the study session.

## Results

The means and standard deviations (SD) of the OSIQ-R scores at baseline and on one-year follow-up are presented in Table 1.

### OSIQ-R total scores

**Figure 1:**
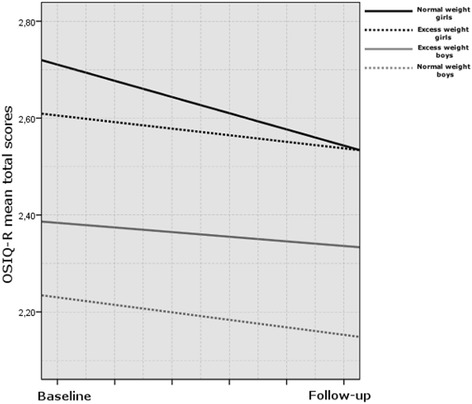
**The Offer Self-Image Questionnaire, Revised (OSIQ-R) mean total scores at baseline and on one-year follow-up among girls with excess weight (n = 38) and those with normal weight (n = 40) and among boys with excess (n = 42) and those with normal weight (n = 46).**

The OSIQ-R mean total scores during the one-year follow-up in the four subgroups are graphically presented in Figure 1. The level differences ((baseline + follow-up)/2) between the groups were significant (overall test p < 0.001), as well as the mean change for all participants (n = 166, p < 0.001). Time*group interaction was also significant (p = 0.012). The change among girls with normal weight was greater than that in other groups (custom contrast, p = 0.001). The other three change scores were very similar. Normal weight girls showed significantly higher change scores than girls with excess weight (p = 0.024) reflecting a more positive development in self-image. The corresponding difference in change scores between normal and excess-weight boys was not statistically significant. Normal weight girls exhibited significantly higher change scores than boys with normal weight (p = 0.048). The corresponding difference between excess-weight girls and boys did not reach statistical significance.

### Component scales

#### Girls

**Figure 2:**
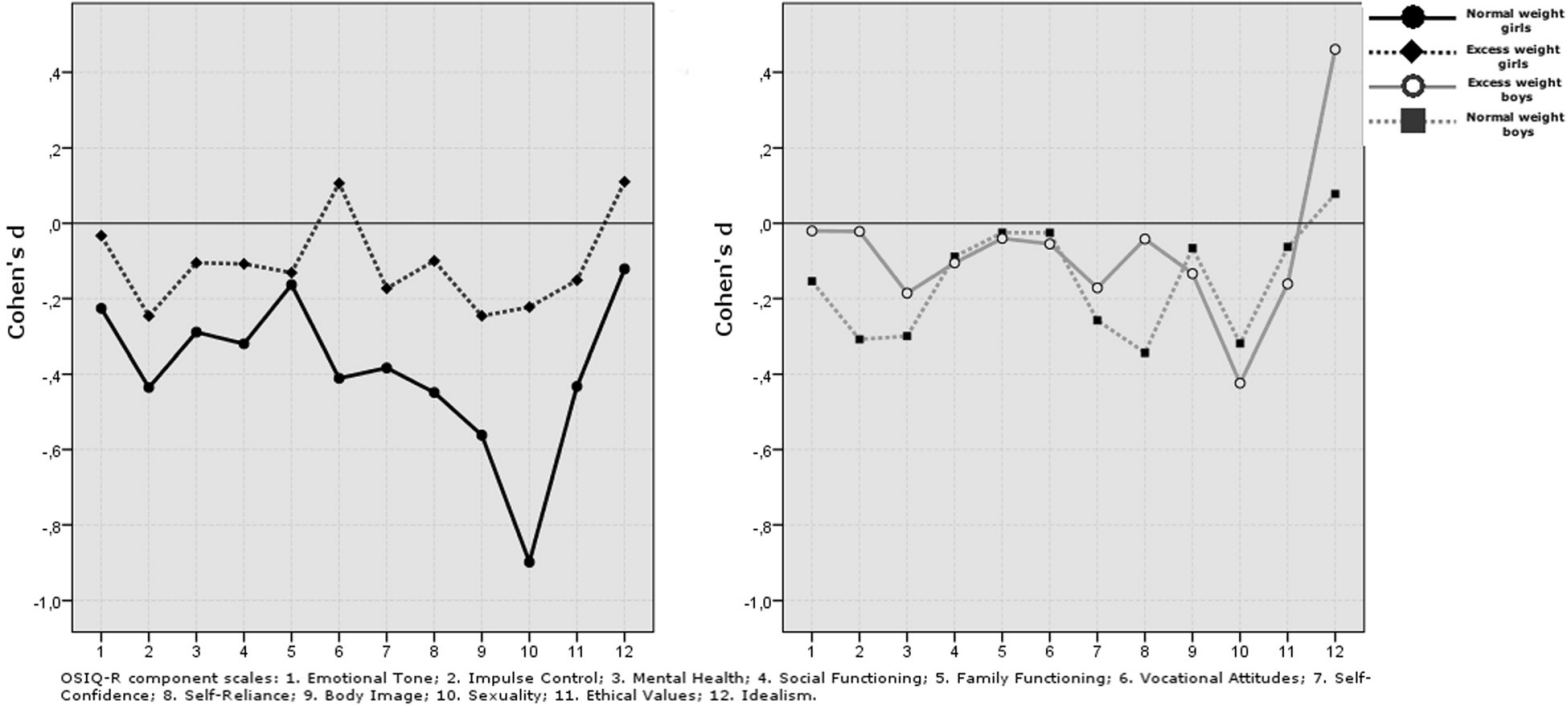
**The Offer Self-Image Questionnaire, Revised (OSIQ-R).** Change scores of the 12 component scales during one-year follow-up among girls with excess weight (n = 38) and those with normal weight (n = 40) and among boys with excess weight (n = 42) and those with normal weight (n = 46).

Among girls with excess weight, most of the change scores in the twelve component scales were negative with the exception of vocational attitudes and idealism, which showed a slightly positive change (Figure 2). The largest change scores compared to zero were recorded for impulse control (d = −0.25), body image (d = −0.25), and sexuality (d = −0.22). However, none of the change scores compared to zero were statistically significant. Among girls with normal weight, all change scores were negative. Significant score changes compared to zero were recorded in impulse control (p = 0.009, d = −0.44), social functioning (p = 0.050, d = −0.32), vocational attitudes (p = 0.013, d = −0.41), self-confidence (p = 0.020, d = −0.38), self-reliance (p = 0.007, d = −0.45), body image (p = 0.001, d = −0.56), sexuality (p < 0.001, d = −0.90), and ethical values (p = 0.009, d = −0.43). In addition, the effect size measured by Cohen’s d was −0.23 and −0.29 in change scores for the components emotional tone and mental health. Girls with normal weight showed significantly higher change scores than those with excess weight in sexuality (p = 0.018, d = −0.52) and vocational attitudes (p = 0.041, d = −0.52). Focusing on effect sizes, girls with normal weight showed a trend to higher change scores than those with excess weight in social functioning (d = −0.24), self-reliance (d = −0.23), ethical values (d = −0.26), and idealism (d = −0.23).

#### Boys

In boys, the change scores were negative with the exception of idealism, which showed positive change scores among both those with excess weight and normal weight (Figure 2).

Among boys with excess weight, change scores compared to zero were significant in sexuality (p = 0.009, d = −0.42) and idealism (p = 0.005, d = 0.46). In boys with normal weight, change scores compared to zero were significant in impulse control (p = 0.042, d = −0.31), mental health (p = 0.048, d = −0.30), self-reliance (p = 0.024, d = −0.34), and sexuality (p = 0.036, d = −0.32). The effect size of change scores was also notable in self-confidence (d = −0.26). When we compared the boys with excess and normal weight, no statistically significant differences emerged in component change scores. However, focusing on effect sizes, boys with normal weight showed a trend to higher change scores than those with excess weight in impulse control (d = −0.27), self-reliance (d = −0.28), and idealism (d = −0.28).

#### Comparisons between girls and boys

When we compared girls and boys with excess weight, no statistically significant differences were observed in the component scores. Focusing on effect sizes, girls showed a trend to higher change scores than boys in impulse control (d = 0.24) and idealism (d = 0.28). When girls and boys with normal weight were compared, girls exhibited significantly higher change scores in sexuality (p = 0.035, d = 0.46) and in body image (p = 0.034, d = 0.52). Using Cohen’s d indices, girls showed a trend to higher change scores in social functioning (d = 0.21), vocational attitudes (d = 0.33), and ethical values (d = 0.28).

#### Dropouts

Among the adolescents with excess weight, the dropouts exhibited significantly higher initial relative weights than those who participated the whole study [42.38 (SD 15.25) vs. 57.20 (SD 17.88); p = 0.040, d = −0.96]. However, no statistically significant difference was observed in BMI, OSIQ-R total or component scores. Among the controls, the dropouts did not significantly differ from those participating in the whole study according to the initial relative weight and BMI. However, they showed significantly higher initial OSIQ-R total scores [2.45 (SD 0.55) vs. 3.07 (SD 0.59); p = 0.017, d = −1.11], as well as component scores in social functioning [2.32 (SD 0.74) vs. 3.26 (SD 0.92); p = 0.008, d = −1.23], family functioning [2.34 (SD 0.77) vs. 3.22 (SD 0.73); p = 0.014, d = −1.16], self-confidence [2.46 (SD 0.61) vs. 3.08 (SD 0.54); p = 0.029, d = −1.02], sexuality [2.51 (SD 0.63) vs. 3.28 (SD 0.64); p = 0.010, d = −1.22], and ethical values [2.55 (SD 0.58) vs. 3.26 (SD 0.57); p = 0.009, d = −1.22].

## Discussion

As far as we are aware, this is the first study to evaluate the development of self-image and its components in non-referred girls and boys with excess and normal weight in mid-adolescence. Most of the research focusing on self-image has been cross-sectional. Adolescence is a development period characterized by intense psychological, emotional, intellectual, and social maturation. This was also observed in our study, and focusing on the regression in OSIQ-R total scores, our finding is in accordance with an earlier study reporting that the change in self-image from early to mid-adolescence in normally developed adolescents is generally positive [16].

For girls with normal weight, mid-adolescence appears to be a period characterized by a rapid development in self-image and its underlying components. Significant score changes compared to zero were observed in impulse control, social functioning, vocational attitudes, self-confidence, self-reliance, body image, sexuality, and ethical values. Among girls with excess weight, there was also a clear trend towards better adjustment, but none of the change scores compared to zero proved to be statistically significant. When the girls with normal and excess weight were compared, the difference in change scores was largest in sexuality and vocational attitudes. Recently, a study investigating self-image among girls in late adolescence with the OSIQ [25] reported that overweight girls were less adjusted with regard to their sexual attitudes than their normal-weight peers. The development of sexual identity already starts to proceed, and intimate and romantic relationships to form in mid-adolescence [2], and from this developmental perspective, our finding is not surprising. Interestingly, the finding is comparable to that reported in girls with severe underweight [20,21]. It appears that for girls, an abnormal weight and body shape easily provokes distress that associates with feelings, attitudes, and behavior towards the opposite sex. Our finding is also in line with earlier research reporting a link between adolescent obesity and problems in forming intimate relationships [11]. Modern Western culture emphasizes thinness [52], and adolescent girls are known to continually compare themselves with their peers, which may lead to severe frustration when a girl sees herself as significantly different from the others. Because of this, overweight girls may experience strong feelings of inadequacy [25]. Furthermore, discriminatory attitudes and behaviors towards obese individuals in education and employment are a reality [53]. This may all reflect in vocational attitudes, including the development of career plans, in girls with excess weight.

Change scores compared to zero were significant among boys with excess weight in sexuality and idealism, and among boys with normal weight in impulse control, mental health, self-reliance and sexuality. When the boys with excess and normal weight were compared, no statistically significant differences emerged in change scores. This finding could be interpreted so that among mid-adolescent boys, the development of self-image varies somewhat depending on the weight status, but is not substantially influenced by overweight and obesity.

One key area of psychological well-being is body image, i.e. the extent to which a person has adjusted to his or her body. There is evidence that obesity is linked to a poor body image, and treatments already exist to improve body image in overweight individuals. Both being female and an early age of onset of obesity have been recognized as risk factors for body image distortions [52]. However, in a cross-sectional OSIQ-R study among normal and overweight girls in late adolescence [25], the difference in body image was not statistically significant. In our study, among girls with normal weight, body image was one of the self-image components with significant change scores towards better adjustment. Among girls with excess weight, body image also showed a change score compared to zero, although this change was not statistically significant. Thus, although development towards better adjustment in body image was observed among these girls, this development was less intense.

From the clinical perspective, it appears that a greater focus on self-image may be indicated in obesity prevention and weight-management programs designed for adolescent populations. Girls with excess weight might particularly benefit from this. Moreover, sexuality, which is one of the key developmental tasks in adolescence [1-3], appears to be more problematic for overweight girls than their healthy peers.

There were some limitations as well as strengths in this follow-up study that need to be mentioned. Although the study involved as many as 24 secondary schools in the city of Helsinki, this represented only one-third of all secondary schools in the capital area. The overall participation rate in the schools was approximately 60%. A nationwide school survey carried out biannually in Finnish comprehensive schools (grades 8 and 9) with the same data collection method has repeatedly reported a participation rate of approximately 80% [54]. Consequently, although the participation rate of the present study cannot be regarded as excellent, it can be considered acceptable. The prevalence of overweight and obesity in adolescence has been reported to vary between 10% and 20% in most European countries [55,56], and consistently with this it was approximately 13% in the present study. An obvious weakness was the limited number of participants with a relative weight of 26% or more above the median. However, a clear strength is that the BMI values were calculated from measurements taken by professional school nurses, since self-reported data are known to underestimate the prevalence of overweight [57]. Nevertheless, those adolescents with the most marked weight problems might have refused to participate in the study because of this methodology. The dropout group consisted of 10 adolescents. The drop-outs in the control group showed more negative self-image than those who attended the whole study, and the drop-outs with excess weight exhibited higher relative weights than those who participated the whole study. However, the impact of the dropouts was very small and did not alter the results or their implications. Sufficient internal consistencies of the components of the OSIQ have been confirmed, except for those components focusing on ethical values and idealism [37,41,58]. In the present study, these same components as well as the self-reliance scale, showed low reliability. Therefore, the results of these three scales must be interpreted with caution. Studies with longer follow-up times are clearly needed in the future.

## Conclusion

In mid-adolescent girls, the influence of overweight and obesity on the development of self-image is substantial. Weight management programs directed at overweight adolescent girls should include psychological interventions aiming to diminish self-image distress, especially that associated with feelings, attitudes, and behavior towards the opposite sex, as well as future career plans.
